# Differential expression and characterization of cypermethrin-degrading potential proteins in *Bacillus thuringiensis* strain, SG4

**DOI:** 10.1007/s13205-016-0541-4

**Published:** 2016-10-19

**Authors:** Pankaj, Geeta Negi, Saurabh Gangola, Priyanka Khati, Govind Kumar, Anjana Srivastava, Anita Sharma

**Affiliations:** 1 Department of Microbiology, College of Basic Sciences and Humanities, G. B. Pant University of Agriculture and Technology, US Nagar, Pantnagar, India; 2 Department of Chemistry, College of Basic Sciences and Humanities, G. B. Pant University of Agriculture and Technology, US Nagar, Pantnagar, India

**Keywords:** Proteomics, Cypermethrin, *Bacillus thuringiensis*, Two-dimensional gel electrophoresis, Biodegradation

## Abstract

A cypermethrin-degrading bacterium (SG4) was isolated from the pesticide-contaminated soil in the agricultural field of the crop research centre of the University, and characterized as *Bacillus thuringiensis* strain, SG4. The bacterium degraded 78.9 % of cypermethrin (50 ppm) in 15 days when grown in a minimal medium. To understand the functional proteins of cypermethrin degradation in *Bacillus thuringiensis* strain SG4, a comparative proteomic analysis was performed in the presence/absence of cypermethrin after 5 days of incubation in minimal medium. More than 450 spots corresponding to different proteins were recorded by 2D electrophoresis. We report expression of 223 and 250 unique proteins under normal and induced conditions (cypermethrin stress), respectively. Identified proteins were categorized into different functional groups on the basis of their biological functions, viz., catabolic enzymes, translational and stress proteins, etc. Characterization of cypermethrin-specific proteins in a bacterial strain will help in biodegradation practices in situ.

## Introduction

Cypermethrin belongs to a synthetic pyrethroid group of insecticides and is used to control insects of cotton and lettuce. It is a broad-spectrum insecticide and kills beneficial insects along with target insects. Fish are particularly more susceptible to cypermethrin. Resistance to cypermethrin (developed quickly in insects exposed frequently to this chemical) renders it ineffective in insects. It readily crosses the blood-brain barrier, enters the brain and eventually leads to nigrostriatal dopaminergic neurodegeneration in rats after prolonged exposure (Tiwari et al. 2010; Singh and Singh 2011; Pankaj et al. 2016). Cypermethrin induces the progressive degeneration of dopaminergic neurons in adults. Degree of neuronal loss is considerably increased when rats are preexposed with low doses of cypermethrin during the critical period of brain development (Singh et al. 2012). Cypermethrin induced nigrostriatal dopaminergic neurodegeneration altering the mitochondrial function was studied using proteomics by Agrawal et al. (2015).

Proteomics has emerged as an excellent approach for gaining insight of physiological changes at cellular level (Jin et al. 2010; Meng et al. 2010) but relatively few attempts have been made to apply this technique to characterize resistance-related proteins in bacteria under pesticide stress. Till date no reports are available analyzing the physiological protein expression in cypermethrin-resistant bacteria in relation to susceptible strain, using proteomics. Proteome-based approaches are effective in making direct links with the pesticide degradation in natural environment. As compared to other biomolecules, proteins are promising markers as they reflect actual functionality with respect to metabolic reactions and regulatory cascades and give direct information about microbial activity than the functional genes and even the corresponding messenger RNAs (Wilmes and Bond 2006). Functional metaproteome from contaminated soil and ground water was analyzed by Benndorf et al. (2007) using 2D electrophoresis. Differential protein expression in response to imidacloprid in *Myzus persicae* (Sulzer) was reported by Meng et al. (2014).

In the present study, combination of two-dimensional gel electrophoresis (2-DE), matrix-assisted laser/desorption ionization time of flight (MALDI–TOF) and mass spectroscopy (MS) were used to identify cypermethrin-resistant proteins in *Bacillus thuringiensis* strain, SG4. A majority of changes in proteins expression are related to signal transduction, RNA processing, protein processing and cypermethrin-degrading catabolic proteins. This study reveals that resistance towards cypermethrin in *Bacillus thuringiensis* strain is a combination of complex responses that are displayed by proteomic expression profiles of resistant bacteria.

## Materials and methods

### Bacterial strain used in the study

Soil samples, collected from pesticide-contaminated agriculture fields of Udham Singh Nagar, Uttarakhand, India, were suspended in minimal medium (MM) containing 50 ppm of cypermethrin in 50 ml medium. After 5 days of incubation, 10 % from the medium was transferred into fresh MM containing cypermethrin@ 50 ppm. Bacterial colonies appeared were transferred individually to 50 ml of minimal medium, supplemented with cypermethrin which acted as a carbon and nitrogen source. Residual concentration of cypermethrin in the presence of bacterial culture was determined by HPLC (Dionex) at different time interval. A pure bacterial isolate showing highest degradation activity was selected for the proteomic study.

### Identification of bacterial strain

Cypermethrin-degrading bacterial isolate SG4 was identified on the basis of morphological, biochemical, and molecular characters (based on 16S rDNA gene sequence). Cell morphology was observed using scanning electron microscopy. Genomic DNA of the bacterial strain was extracted according to Bazzicalupo and Fani (1995). 16S rDNA gene was amplified as described by Negi et al. (2014). PCR product was purified using Genei gel purification kit and sequenced by Biotech Centre, Delhi University, South Campus. Resulting 16S rDNA gene sequences were compared with the sequences in the GenBank nucleotide library using BLAST program (Pankaj et al. 2016). Multiple sequence alignment was carried out using Clustal-W and phylogeny was analyzed using MEGA 5.0 software Pankaj (2015).

### Sample preparation for 2D gel electrophoresis

Whole cell extracellular differential proteome with and without cypermethrin was extracted from an actively growing bacterial isolate (SG4) after 5 days of incubation in minimal medium. The concentration of the pesticide was 200 ppm. Extraction procedure and methodology for PAGE analysis were followed as described by Laemmli (1970). Protein samples were lyophilized at Department of Molecular Biology and Genetic Engineering. GBPUAT, Pantnagar. Lyophilized protein samples were sent to Sandor life Science Pvt. Ltd., Hyderabad for 2D gel electrophoresis.

### Gel image analysis

In silico analysis of 2D gel was performed on the basis of isoelectric point (pI) and molecular weight of separated protein(s) as reported by Jain et al. (2010). ExPASy is a SIB Bioinformatics Resource Portal which provides access to scientific databases and software tools in proteomics, genomics, phylogeny, systems biology and transcriptomics, etc. We have used TagIdent (a software tool) to analyze stress proteins that appeared as prominent spots in two-dimensional gel electrophoresis in SG4 exposed to pesticide.

### MALDI–TOF–MS analysis and MASCOT database searches

MALDI–TOF analysis of match ID 2 and 42 was done at Sandor Proteomics Pvt Ltd, Hyderabad using Bruker Daltonics—UltraflexTM III Mass Spectrometer with spectra internally calibrated using trypsin auto-digestion products. Obtained peptide masses were searched on MascotTM Peptide Mass Fingerprint database (Matrix Science). Obtained data was used to identity the proteins using Mascot search tool (http://www.matrixscience.com).

## Results

### Isolation and identification of cypermethrin-degrading strain

**Fig. 1:**
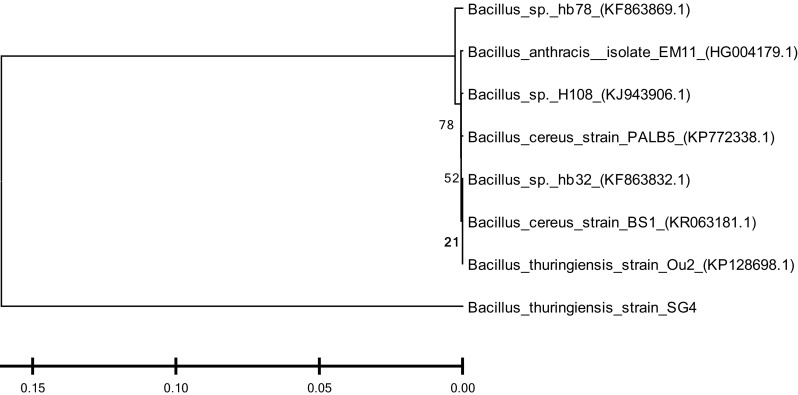
Phylogenetic analysis of SG4 strain on the basis of 16S rDNA using MEGA 5.0 software. The *numbers* in *parentheses* represent the sequence accession number in GenBank. *Bar* represents sequence divergence

**Fig. 2:**
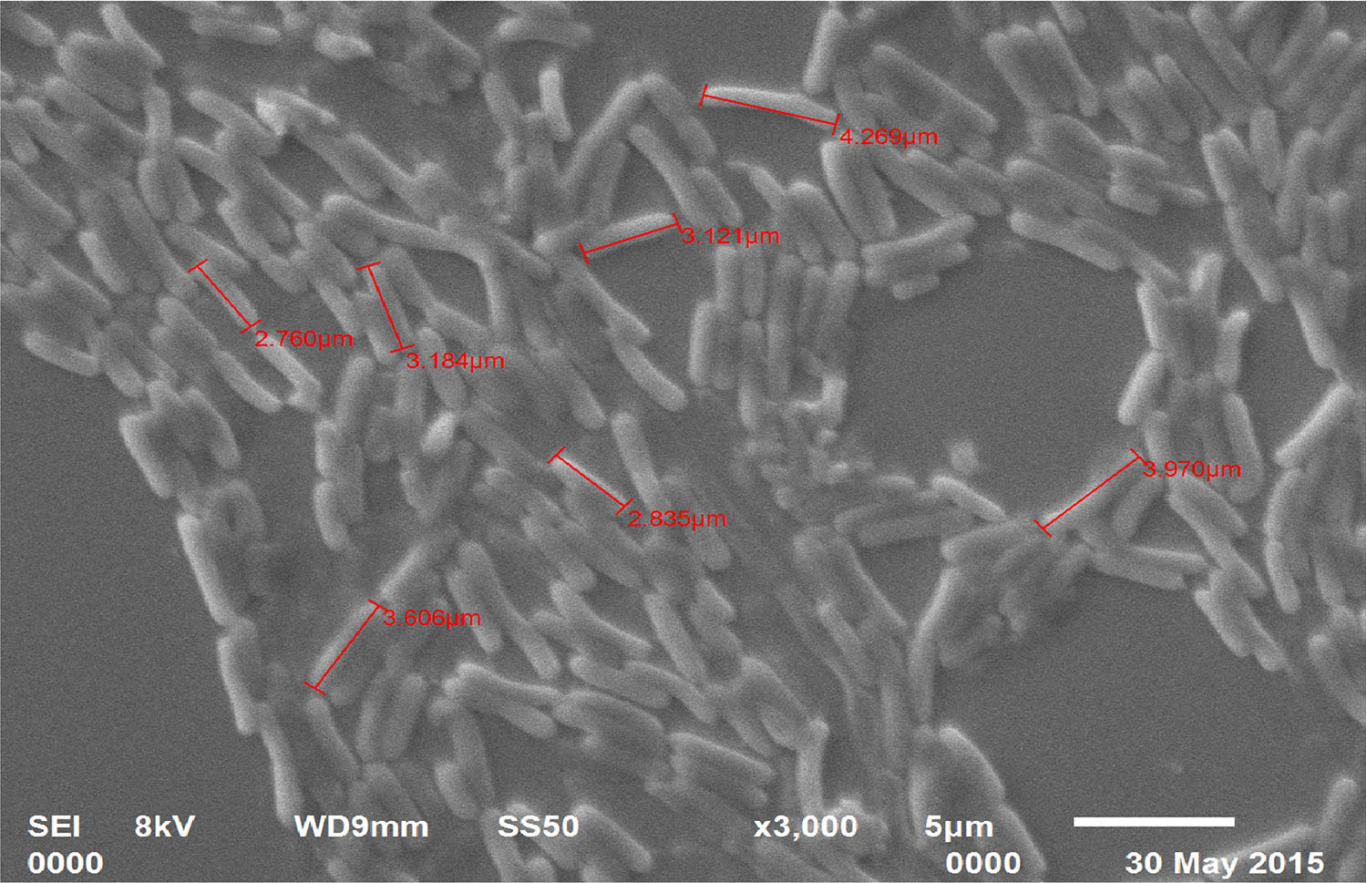
Electron micrograph of strain SG4 with cypermethrin

**Fig. 3:**
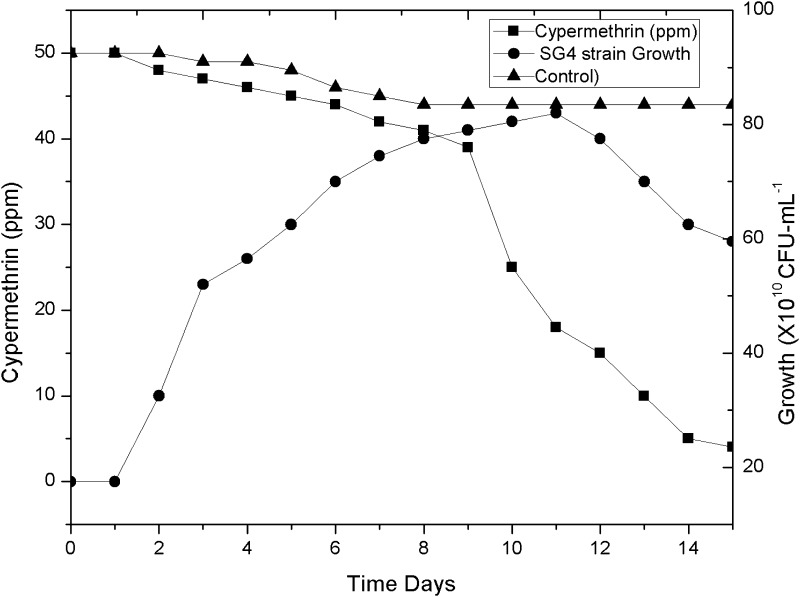
Growth-linked biodegradation of cypermethrin by SG4

A highly efficient, cypermethrin-degrading bacterial strain, designated as SG4, was isolated from pesticide-contaminated agriculture fields of Udham Singh Nagar using enrichment method. The organism utilized cypermethrin as a carbon and nitrogen source when grown in minimal medium and degraded the compound by 78.9 % within 15 days (Fig. 3). Bacterial isolate was Gram-positive and rod-shaped (Fig. 2). Phylogenetic analysis of 16S rDNA gene sequences revealed that the strain showed 99–100 % homology with *Bacillus thuringiensis* (Fig. 1). 16S rDNA sequences of *Bacillus thuringiensis* strain SG4 were deposited in GenBank under the nucleotide accession number, KT186610.

### 2-DE analysis of proteins in *Bacillus thuringiensis* strain SG4

**Fig. 4:**
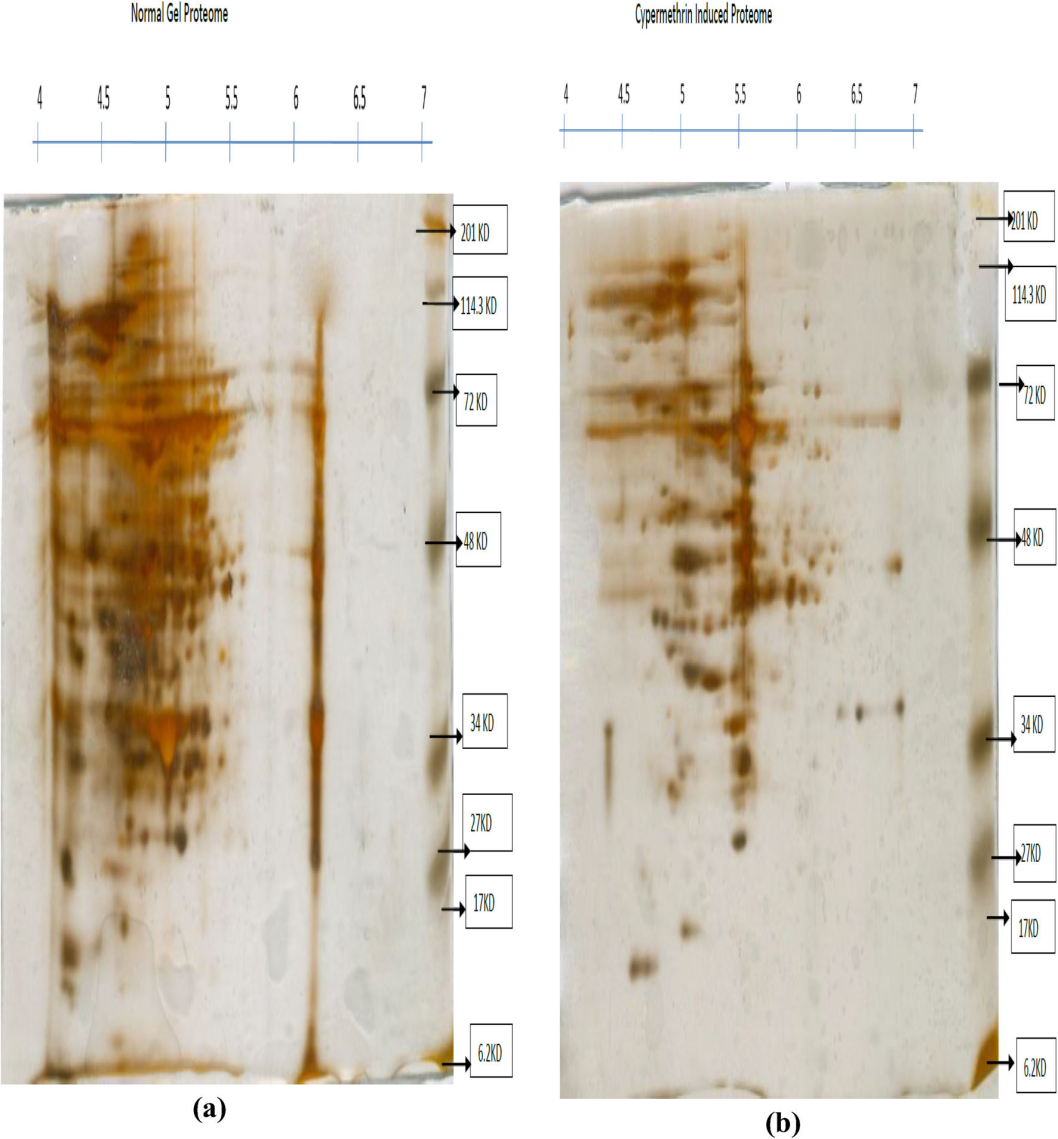
Two-dimensional gel electrophoresis of cypermethrin-induced proteins in SG4. **a** Normal protein profile without cypermethrin. **b** Cypermethrin-induced protein

**Fig. 5:**
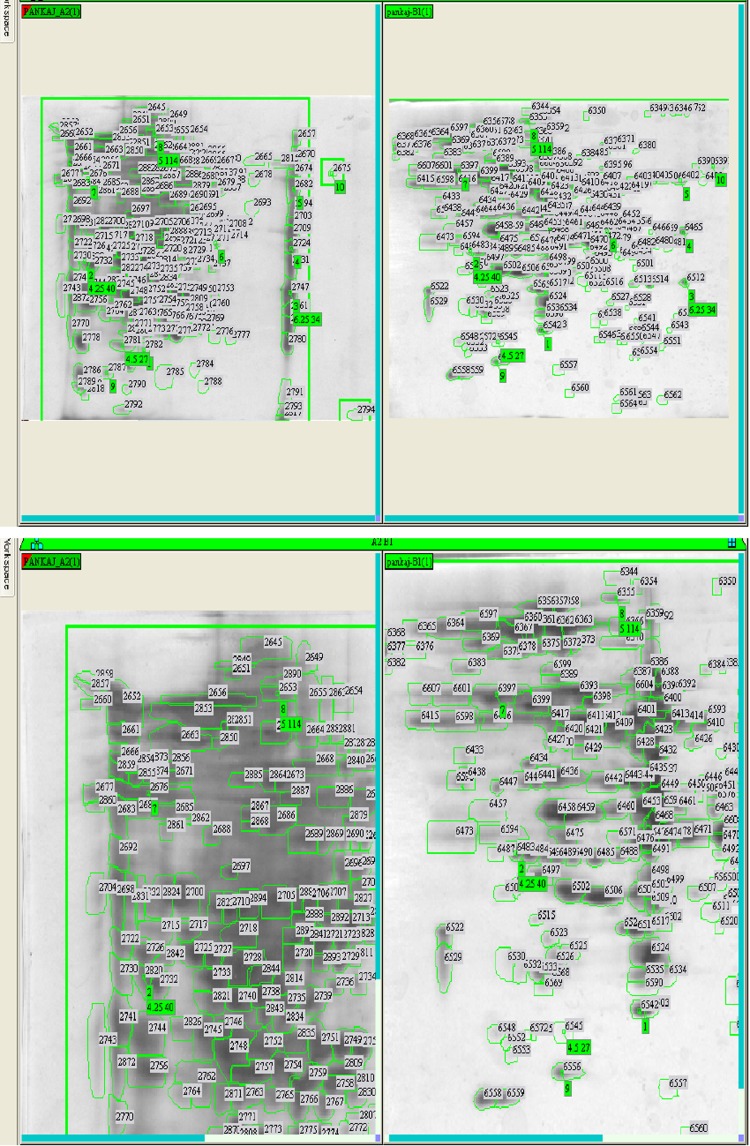
Comparative 2-D profiling on the basis of observed spots

**Fig. 6:**
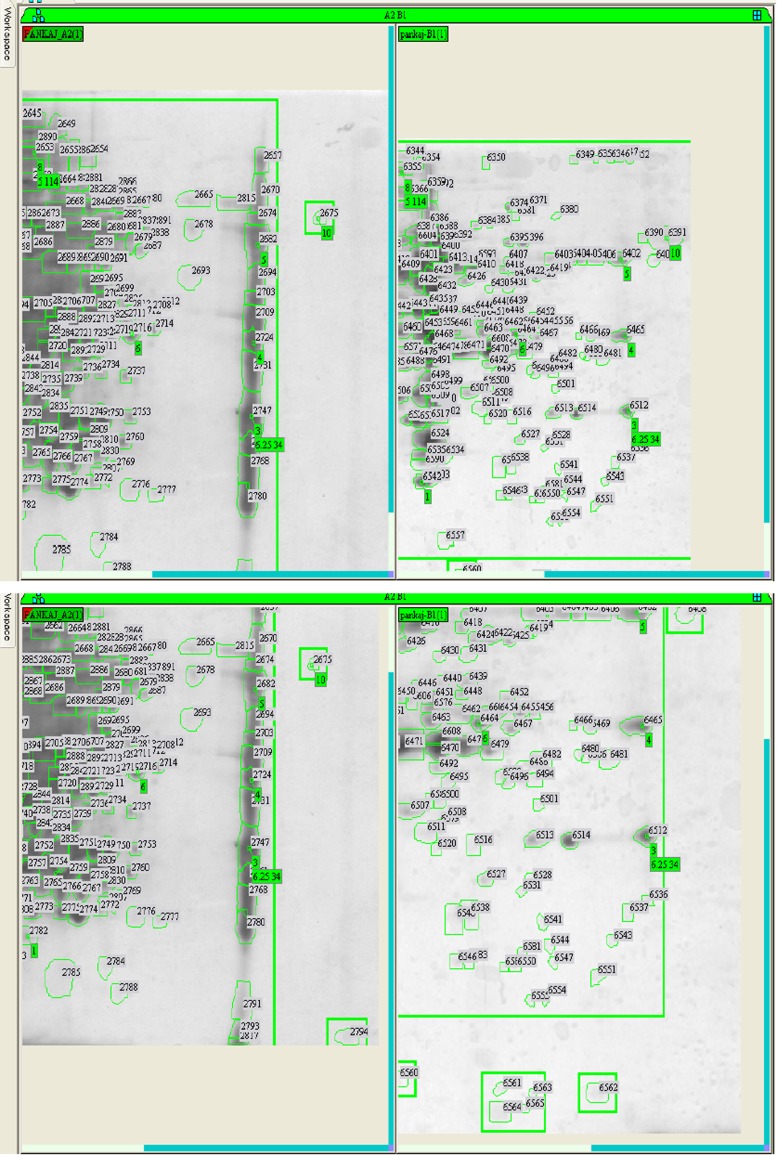
Comparative 2-D profiling of expressed proteins on the basis of observed spots and identification of unique spots

**Fig. 7:**
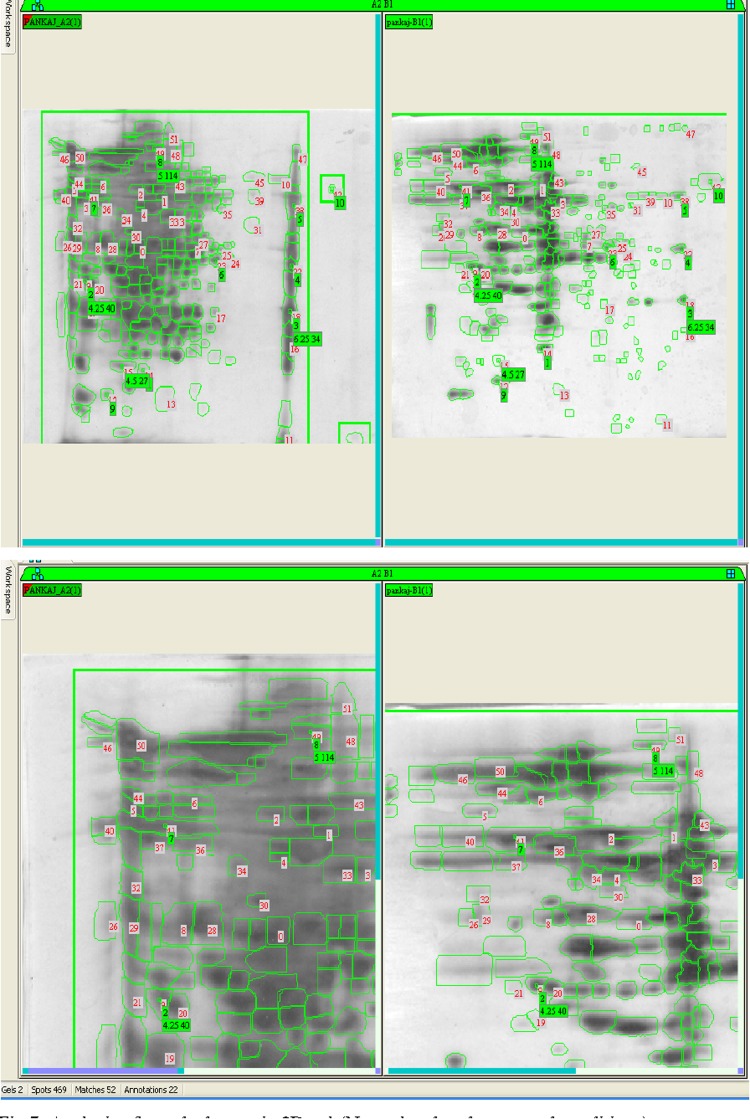
Analysis of matched spots in 2D gel (normal and under stressed conditions)

**Fig. 8:**
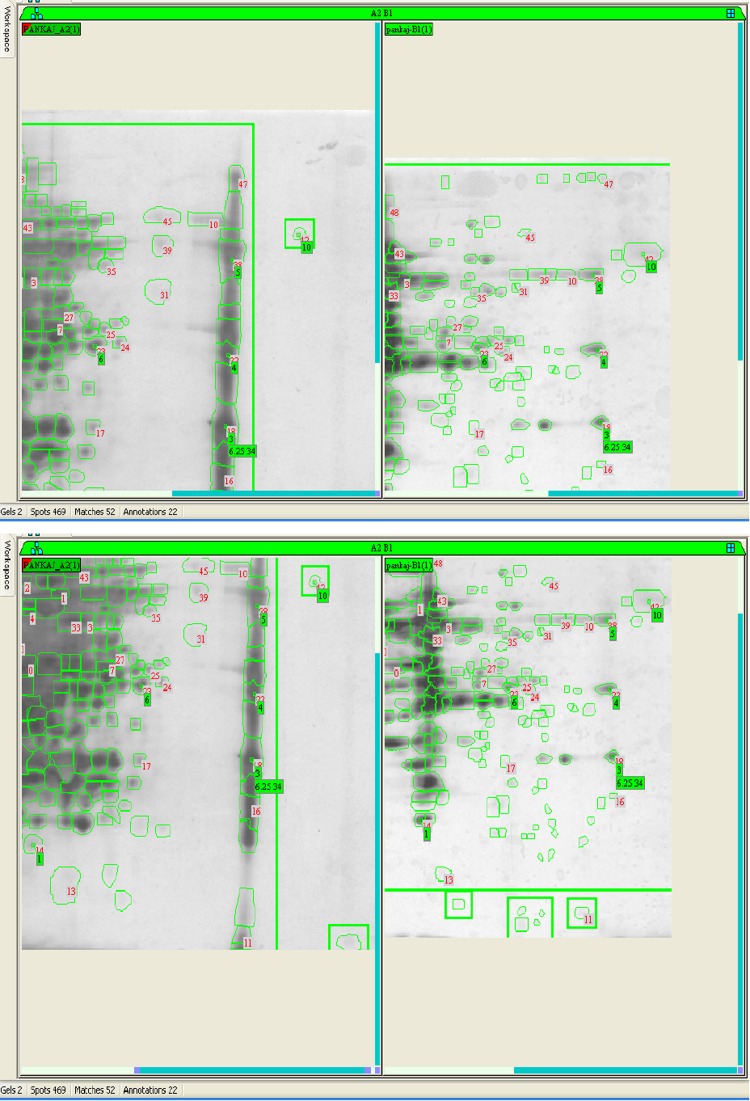
Analysis of matched spots in 2D gel (down-regulation)

Difference in protein expression in any kind of stress (susceptible and resistant) in bacterial strains is observed during physiological adaptations. Protein samples of SG4 (extracted from SG4, exposed to cypermethrin and one control) were subjected to two-dimensional gel electrophoresis (Fig. 4a, b). Representative 2D maps are shown in Figs. 5, 6, 7 and 8. Separated proteins spots are evident in both the dimensions. Gel maps were of good quality and could be used as reference 2-D maps for *Bacillus thuringiensis* strain SG4 proteins. More than 450 protein spots were reproducibly detected by Image analysis software. Quantitative image analysis revealed the presence of 51 protein spots that showed significant and reproducible changes in protein expression under pesticide stress (Figs. 6, 7). Some proteins were overexpressed and others were underexpressed. Magnified view of differentially expressed proteins is given in Fig. 8.

### Identification of differentially expressed proteins

**Fig. 9:**
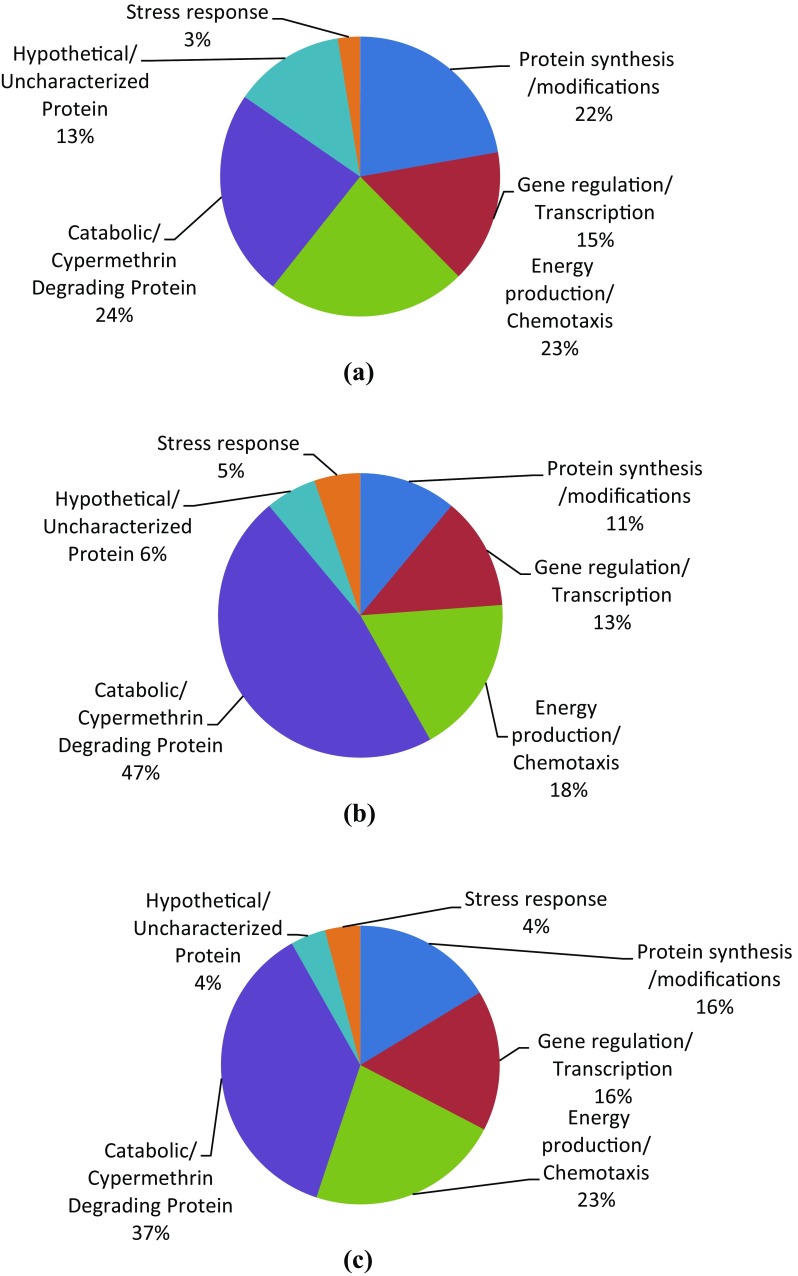
**a** Distribution of proteins expressed during normal metabolism in SG4. **b** Distribution of proteins expressed during cypermethrin-induced metabolism in SG4. **c** Overexpressed and underexpressed proteins in cypermethrin-induced condition in strain SG4

Differentially expressed proteins in SG4 were identified on the basis of their PI values and molecular weight using ExPASy protein database. Highly up-regulated proteins were identified using MALDI–TOF mass spectrometer. Out of 51 differentially expressed proteins, 39 protein spots showed apparent similarities with database, including 21 down-regulated spots and 18 up-regulated spots in resistant *Bacillus thuringiensis* strain SG4 (Table 1). Remaining 12 proteins did not show major changes statistically. We report the expression of 223 unique proteins in SG4 under normal conditions and 250 unique proteins under cypermethrin-induced conditions. Majority of the spots in 2D gels displayed a reasonable correlation of theoretical molecular weight (MW) values with the experimental data. Some of spots showed significant deviation from the experimentally measured MW from the expected theoretical values, implying that PTMs (post translational modifications) such as fragmentation might had occured in these proteins (Liu et al. 2008). Most of the theoretical values were based on proteins originating from other species (rather than *Bacillus thuringiensis*), rendering the exact MW of these proteins unknown. Based on the biological functions, identified proteins were classified into several functional categories including Stress response, hypothetical/uncharacterized protein, catabolic/cypermethrin-degrading proteins, protein synthesis/modifications, gene regulation/transcription, energy production/chemotaxis etc. Hypothetical proteins and some proteins with unknown function were also identified in the database (Fig. 9a–c).

**Table 1:** Overexpressed and underexpressed proteins in cypermethrin-induced SG4 strain

Overexpressed spots		Protein	Underexpressed spots		Protein
Match ID	Fold increase		Match ID	Fold decrease	
41	1.67678755	Putative Gly-rich membrane protein Bcell_0380	16	0.021785225	Pantothenate synthetase
39	1.77924122	Polyribonucleotide nucleotidyltransferase	19	0.05133814	Ribosomal protein L11 methyltransferase
12	1.77953783	Protein phosphatase CheZ	47	0.05781404	Formate dehydrogenase
36	1.83405620	Threonine–tRNA ligase	45	0.09285401	Protein translocase subunit SecA
44	1.85478053	Poly(beta-d-mannuronate) C5 epimerase 7	31	0.12415845	Dihydroxy-acid dehydratase
34	2.10710685	DNA mismatch repair protein MutL	18	0.14729877	Glycerol-3-phosphate dehydrogenase [NAD(P)+]
40	2.22745952	Catalase-peroxidase	15	0.18077879	Hydroxyethylthiazole kinase
30	2.26218743	Carbamoyl-phosphate synthase large chain, C-terminal section	27	0.18420540	Phosphoenolpyruvate carboxykinase [ATP]
3	2.26840416	Chaperone protein HscA	11	0.21590603	Peptide deformylase
33	2.28367285	Chaperone protein DnaK	21	0.22245105	3-Hydroxy-3-methylglutaryl-coenzyme A reductase
5	2.29104343	UPF0753 protein pNG7034	13	0.22305841	3-Isopropylmalate dehydratase small subunit
14	2.83744844	UDP-2,3-diacylglucosamine hydrolase	8	0.33405703	Alpha-amylase 1
6	3.69354763	Type 2 DNA topoisomerase 6 subunit B	38	0.33998292	Isocitrate dehydrogenase kinase/phosphatase
0	4.18907236	Glutamyl-tRNA(Gln) amidotransferase subunit A	29	0.36236346	3-Phosphoshikimate 1-carboxyvinyltransferase
46	4.87552001	Uncharacterized PPE family protein PPE24	32	0.36627301	Alpha-ketoglutarate semialdehyde dehydrogenase
4	11.3700073	Chaperone protein DnaK	17	0.43840827	Malate dehydrogenase
42	16.6782931	Ribonucleoside-diphosphate reductase subunit alpha	7	0.45626644	NADH-quinone oxidoreductase subunit N
2	18.2576219	RNA polymerase II subunit A C-terminal domain phosphatase	25	0.46624517	Putative cysteine ligase BshC
			22	0.48230177	NADH-quinone oxidoreductase subunit D
			26	0.49689944	Dihydrolipoyl dehydrogenase
			51	0.49926538	DNA-directed RNA polymerase subunit beta 1

All the values are significant at *p* < 0.05

## Discussion

Cypermethrin is used widely against serious pests of most of the crops worldwide. Extensive use of number of pesticides has resulted in rapid development of insecticide resistance in naturally occurring microbiota (Meng et al. 2014; Foster et al. 2003; Slater et al. 2011). Development of resistance against an insecticide in a bacterial system is an universal phenomena. A comparative proteomic analysis of susceptible and resistant bacteria is helpful to elucidate innate responses and adaptive physiological changes contributing towards cypermethrin resistance. The goal of the present study was to identify and characterize cypermethrin responsive proteins in a cypermethrin-resistant *Bacillus thuringiensis* strain SG4 using proteomic approach. Differentially expressed protein (51 spots in gel) were observed in resistant *Bacillus thuringiensis* strain SG4 in comparison to the same strain growing without cypermethrin stress. Total number of protein spots was 223 in normal condition whereas 250 in cypermethrin induced conditions. Most of proteins were found to have important biological functions, providing a basis for understanding and annotating their role in response to cypermethrin exposure. Identified proteins could be classified into six groups: stress response, hypothetical/uncharacterized protein, catabolic/cypermethrin-degrading proteins, protein synthesis/modifications, gene regulation/transcription, energy production and chemotaxis-related proteins.

### Stress response related proteins

Expression of functional proteins through signal transduction and regulation of gene expression might work cooperatively to maintain cellular homeostasis under various external conditions. In this study some chaperones were identified, including molecular chaperone DnaK (spot 2868), DNA repair protein MutL (spot 34), chaperone proteinHsc A (spot 2869), Grp E (spot 6559),with one homolog (spot 6421) and chaperone protein HtpG (spot 6401). HscA is a heat shock protein whose synthesis is stimulated under stress (Pumirat et al. 2009). It is known that oxidative stress can be induced by pesticides either by overproduction of free radicals or by alterations in antioxidant defence mechanism (Abdollahi et al. 2004). Catalase (CAT) is one of the essential enzymes in the process of reactive oxygen species (ROS) detoxification (Gao et al. 2008). Our results showed that predicted catalase activity was inhibited in cypermethrin-resistant bacteria. Inhibition of catalase activity could be explained by level of toxicity of cypermethrin or by the presence of O^2^ radicals during resistance development. Several studies have demonstrated that higher quantity of O^2−^ radicals acts as catalase inhibitor, so it is possible that exposure to cypermethrin during the development of resistance, primarily induced the generation of O^2−^ radicals which caused inhibition of the expression of CAT. Other studies have also reported inhibition of CAT activity after exposure to some pesticides (Ferrari et al. 2011; Velki and Hackenberger 2012).

### Cypermethrin-degrading proteins

Metabolic pathways produce energy for the maintenance of bacterial growth under stress condition. Primary metabolic pathways (metabolism of carbohydrate, fatty acid for energy production) are needed to stabilize a new homeostasis in the resistant bacteria. Some enzymes like malate dehydrogenase, α-ketoglutarate semialdehyde dehydrogenase, isocitrate dehydrogenase kinase/phosphatase, glycerol-3- phosphate dehydrogenase and formate dehydrogenase were down-regulated in cypermethrin-resistant bacteria. All the mentioned enzymes have significant role in tri-carboxylic acid cycle suggesting that bacteria might have taken up cypermethrin through novel metabolites for its growth. Such findings were not reported earlier in the similar studies conducted by Mishra and Shukla (2003). Reduction in malate dehydrogenase activity in *Clarias batrachus* under endosulfan stress was reported by Mishra and Shukla (2003) and it could be due to binding of endosulfan or its metabolites with enzyme molecules and/or by blocking the enzyme synthesis.

### Protein synthesis/modifications

Protein synthesis can be regulated by modifications in various enzymes at post transcriptional, translational and post translational level. Present study reports the involvement of different up and down-regulated proteins in these processes and mentions the up-regulation of polynucleotide nucleotidyltransferase (Match ID39), Threonine-tRNA ligase (Match ID 36), Glutaryl-tRNA (Gln) amidotransferase subunit A, (match ID 0), and RNA polymerase II subunit A C-terminal domain phosphatise (Match ID 2), whereas the DNA-directed RNA polymerase (β 1 subunit, Match ID 51) was down-regulated.

### Gene regulation/transcription

Gene expression can be regulated at replication, transcriptional and translational level. Proteins involved in all these processes were identified in control and cypermethrin-resistant bacteria. Overexpressed proteins were DNA mismatch repair protein MutL (Match ID 34) and type 2 DNA topoisomerase 6 subunit B (Match ID 6). Besides these, several unique proteins were also expressed positively. Some important proteins were: elongation factor 4 (spot 2833), protein RecA (spot 2739), transcription factor TBF1 (spot 2852), elongation factor Ts (spot 2767), peptide chain release factor 1 (spot 2736) and cysteine ligase BshC (spot 2712) (Table 1).

### Energy production/chemotaxis

Chemotaxis, a chemical interaction between the chemicals and bacterial cell surface receptors plays important role in energy production. A number of proteins take part in energy production and chemotaxis. In the present study, Phosphatise CheZ (Match ID 12) and putative glycine rich membrane protein (Match ID 41) were overexpressed in cypermethrin-resistant bacteria. Whereas translocase subunit Sec A protein (match ID 45) was down-regulated under cypermethrin induction in *Bacillus thuringiensis* strain SG4. Other prominent proteins in resistant bacteria were NAD kinase (6572), putative arsenical pump driving ATPase (6598), chemotaxis response regulator glutamate methyl transferase (6570), ATPase synthase (subunit α 6437) and ATPase synthase subunit β (6449), etc.

## Conclusion

The present study offers an example of application of proteomic approach to screen cypermethrin resistance proteins in *Bacillus thuringiensis* strain, SG4. Response of *Bacillus thuringiensis* strain, SG4 towards cypermethrin is a complex phenomenon, as differentially expressed proteins are involved in multiple functional categories. Most of the altered proteins were closely associated with stress response, hypothetical/uncharacterized proteins, catabolic/cypermethrin-degrading proteins, protein synthesis/modifications, gene regulation/transcription, energy production and chemotaxis-related proteins. But identifying all the proteins in insecticide-resistant bacteria is not sufficient to reveal the complexity underlying the engineered object. The proteomic resources and knowledge developed by this study will contribute towards better understanding of the mechanisms which govern development of insecticide resistance in bacterial systems.
